# Longitudinal Reliability of Milestones-Based Learning Trajectories in Family Medicine Residents

**DOI:** 10.1001/jamanetworkopen.2021.37179

**Published:** 2021-12-07

**Authors:** Yoon Soo Park, Stanley J. Hamstra, Kenji Yamazaki, Eric Holmboe

**Affiliations:** 1 Harvard Medical School, Boston, Massachusetts; 2 Massachusetts General Hospital, Boston; 3 University of Illinois at Chicago College of Medicine, Chicago; 4 Accreditation Council for Graduate Medical Education, Chicago, Illinois; 5 Department of Surgery, University of Toronto, Toronto, Ontario, Canada; 6 Feinberg School of Medicine, Northwestern University, Chicago, Illinois

## Abstract

**Importance:**

Longitudinal Milestones data reported to the Accreditation Council for Graduate Medical Education (ACGME) can be used to measure the developmental and educational progression of learners. Learning trajectories illustrate the pattern and rate at which learners acquire competencies toward unsupervised practice.

**Objective:**

To investigate the reliability of learning trajectories and patterns of learning progression that can support meaningful intervention and remediation for residents.

**Design, Setting, and Participants:**

This national retrospective cohort study included Milestones data from residents in family medicine, representing 6 semi-annual reporting periods from July 2016 to June 2019.

**Interventions:**

Longitudinal formative assessment using the Milestones assessment system reported to the ACGME.

**Main Outcomes and Measures:**

To estimate longitudinal consistency, growth rate reliability (GRR) and growth curve reliability (GCR) for 22 subcompetencies in the ACGME family medicine Milestones were used, incorporating clustering effects at the program level. Latent class growth curve models were used to examine longitudinal learning trajectories.

**Results:**

This study included Milestones ratings from 3872 residents in 514 programs. The Milestones reporting system reliably differentiated individual longitudinal patterns for formative purposes (mean [SD] GRR, 0.63 [0.03]); there was also evidence of precision for model-based rates of change (mean [SD] GCR, 0.91 [0.02]). Milestones ratings increased significantly across training years and reporting periods (mean [SD] of 0.55 [0.04] Milestones units per reporting period; *P* < .001); patterns of developmental progress varied by subcompetency. There were 3 or 4 distinct patterns of learning trajectories for each of the 22 subcompetencies. For example, for the professionalism subcompetency, residents were classified to 4 groups of learning trajectories; during the 3-year family medicine training period, trajectories diverged further after postgraduate year (PGY) 1, indicating a potential remediation point between the end of PGY 1 and the beginning of PGY 2 for struggling learners, who represented 16% of learners (620 residents). Similar inferences for learning trajectories were found for practice-based learning and improvement, systems-based practice, and interpersonal and communication skills. Subcompetencies in medical knowledge and patient care demonstrated more consistent patterns of upward growth.

**Conclusions and Relevance:**

These findings suggest that the Milestones reporting system provides reliable longitudinal data for individualized tracking of progress in all subcompetencies. Learning trajectories with supporting reliability evidence could be used to understand residents’ developmental progress and tailored for individualized learning plans and remediation.

## Key Points

**Question:**

Can Milestones ratings be used to create reliable learning trajectories for resident physicians that identify developmental growth patterns toward unsupervised practice?

**Findings:**

This cohort study of 3872 family medicine residents found that the Milestones assessment system had high reliability for measuring the developmental growth of learners. Each family medicine subcompetency included 3 or 4 distinct patterns of learning trajectories that could be used to support learner feedback.

**Meaning:**

These findings suggest that identifying different patterns of learning trajectories using the Milestones assessment system could support and provide early remediation for struggling learners who may not meet graduation targets in training.

## Teaser

This cohort study investigates the reliability of learning trajectories and patterns of learning progression that can support meaningful intervention and remediation for residents.

## Introduction

Decisions for promotion and readiness for unsupervised practice in graduate medical education require ongoing monitoring of learner performance using robust and longitudinal assessment systems data.^1,2^ These aspirations to use ongoing assessment data form an integral aspect of competency-based medical education from a developmental perspective, to identify areas for early remediation and to facilitate further growth for all learners, meeting goals at critical levels for transition to subsequent stages of training.^3,4^

The Accreditation Council for Graduate Medical Education (ACGME) implemented Milestones through the Next Accreditation System (NAS) initiative in July 2013. As part of NAS, resident progress is tracked using a developmental model through the achievement of milestones within specialty-specific subcompetencies.^5^ Milestones are developmental levels defined in more granular narrative terms across the 6 ACGME Core Competencies, which residents are expected to learn and demonstrate. Every 6 months, the residency program’s Clinical Competency Committee (CCC) synthesizes assessment data into Milestone levels, which the program subsequently reports to the ACGME.^6,7,8^ Validity evidence supporting the use and interpretation of Milestones data warrants further investigation, particularly from a longitudinal and developmental viewpoint of assessment data.^9,10,11,12,13,14^

Longitudinal assessments can be described as learning trajectories to measure the developmental progression of learners.^15,16,17,18^ Learning trajectories represent longitudinal patterns of developmental progress, measuring growth and acquisition of competencies over time.^15,16^ In this regard, identifying meaningful patterns that reveal inflection points in learner’s developmental progress and examining factors influencing the variability of milestones level (eg, program-level effects) can be informative. Although different patterns of developmental progress may exist, a learner may improve consistently during the initial phase of training but plateau at later stages; learners may also progress with varying inflection points (shift in the direction of the slope) during training when their performance stagnates or decreases.^18^ Prior work by Holmboe et al^19^ began examining Milestones data at the national level to help inform predictive analytics. Identifying groups of such patterns may facilitate developing individual learning plans, which can serve to target and provide early remediation for learners who may show signs of difficulty in meeting their graduation targets.

In this study, we used national longitudinal cohort data of family medicine residents from entry to graduation to examine the longitudinal reliability of Milestones data and to explore their learning trajectories toward unsupervised practice. We built on the validity of Milestones-based data to support their use as learner analytics, focusing on the internal structure validity evidence (longitudinal reliability and variance components at the learner and program levels) and learning trajectories (patterns of developmental growth and points of potential remediation).^17,20,21,22,23^ We aim to identify the reliability of learning trajectories to detect differential progression that can yield meaningful results at the residency program level as well as at the learner level.

## Methods

### Study Design, Setting, and Participants

We use national retrospective longitudinal cohort data of family medicine residents who entered training in July 2016 (postgraduate year [PGY] 1) and graduated in June 2019 (PGY 3). Milestones assessment data of residents are gathered by the ACGME as part of standard education and accreditation purposes. Therefore, the institutional review board at the American Institutes for Research granted exempt status to this study and waived the requirement for informed consent. This study followed the Strengthening the Reporting of Observational Studies in Epidemiology (STROBE) reporting guideline.^24^ We used national cohort data from 514 residency programs and 3872 residents in family medicine who reported their Milestones data to the ACGME between 2016 and 2019.

### Variables

#### Family Medicine Milestones

Family medicine is a 3-year training program with 22 subcompetencies across the 6 ACGME Core Competencies; there are 5 patient care (PC) subcompetencies, 2 medical knowledge (MK) subcompetencies, 3 practice-based learning and improvement (PBLI) subcompetencies, 4 system-based practice (SBP) subcompetencies, 4 professionalism subcompetencies, and 4 interpersonal communication skills (ICS) subcompetencies (Table 1).^25^ Validity evidence supporting Milestones content and its development process has been described previously.^26^

**Table 1.** Longitudinal Reliability and Variance at the Learner- and Program-Level for 3872 Learners in 514 Programs

ACGME core competency and family medicine subcompetencies	Longitudinal reliability^a^		Random-effects variance, %^b^			
	Growth rate	Growth curve	Learner intercept	Program intercept	Learner slope	Program slope
PC						
PC-1: cares for acutely ill or injured patients in urgent and emergent situations and in all settings	0.64	0.93	25	34	4	7
PC-2: cares for patients with chronic conditions	0.63	0.94	22	35	4	8
PC-3: partners with the patient, family, and community to improve health through disease prevention and health promotion	0.64	0.93	22	36	4	8
PC-4: partners with the patient to address issues of ongoing signs, symptoms, or health concerns	0.62	0.93	23	33	5	7
PC-5: performs specialty-appropriate procedures and is knowledgeable about procedures performed by other specialists	0.60	0.92	23	33	5	7
MK						
MK-1: demonstrates medical knowledge of sufficient breadth and depth to practice family medicine	0.54	0.90	23	31	2	7
MK-2: applies critical thinking skills in patient care	0.63	0.92	26	32	5	6
SBP						
SBP-1: provides cost-conscious medical care	0.63	0.93	21	36	4	7
SBP-2: emphasizes patient safety	0.59	0.92	18	35	4	9
SBP-3: advocates for individual and community health	0.63	0.91	18	37	4	9
SBP-4: coordinates team-based care	0.62	0.91	23	34	4	7
PBLI						
PBLI-1: locates, appraises, and assimilates evidence from scientific studies related to the patients’ health problems	0.65	0.92	20	35	4	9
PBLI-2: demonstrates self-directed learning	0.63	0.91	24	34	4	7
PBLI-3: improves systems in which the physician provides care	0.60	0.90	17	36	4	9
Professionalism						
Professionalism-1: completes a process of professionalization	0.65	0.88	21	37	4	7
Professionalism-2: demonstrates professional conduct and accountability	0.61	0.86	20	34	6	7
Professionalism-3: demonstrates humanism and cultural proficiency	0.67	0.90	21	38	4	7
Professionalism-4: maintains emotional, physical, and mental health and pursues continual personal and professional growth	0.62	0.89	21	35	5	7
ICS						
ICS-1: develops meaningful, therapeutic relationships with patients and families	0.68	0.91	23	36	5	7
ICS-2: communicates effectively with patients, families, and the public	0.65	0.91	22	36	4	7
ICS-3: develops relationships and effectively communicates with physicians, other health professionals, and health care teams	0.66	0.90	23	35	5	8
ICS-4: utilizes technology to optimize communication	0.66	0.89	19	38	4	8

Abbreviations: ACGME, Accreditation Council for Graduate Medical Education; ICS, interpersonal and communication skills; MK, medical knowledge; PC, patient care; PBLI, practice-based learning and improvement; SBP, system-based practice.

^a^ Growth rate reliability is used to make inferences about individual growth differences; growth curve reliability is used to make model-based inferences, including the longitudinal statistical methods used in this study. Both statistics quantify the proportion of information relative to construct irrelevant variance across the longitudinal educational data used to make inferences.

^b^ Learner intercept and program intercept indicate the percentage of variability at baseline (start of training) for learners and programs, respectively; learner slope and program slope indicate the percentage of variability in growth for learners and programs, respectively. Complete parameter estimates and associated results appear in the eTable in the Supplement.

The Milestones data are on a 10-point scale between level 1 and level 5 in 0.50-unit intervals (and also a preceding level 0 to indicate that the leaner has not achieved level 1). Level 4 is specified as the recommended graduation target, indicating readiness for unsupervised practice. Milestones are designed for mainly formative purposes and are not used for accrediting individual residency programs or eligibility determinations for board certification.

#### Internal Structure Validity Evidence: Longitudinal Reliability and Variability of Milestones Ratings

Growth rate reliability (GRR) and growth curve reliability (GCR)^11,12,13^ are standard techniques for measuring the consistency of assessment data over time, quantifying the proportion of information (signal) relative to construct irrelevant variance (error) in longitudinal educational data to reflect meaningful longitudinal trends. If the intended purposes of longitudinal assessment data are to make inferences about individual growth differences, the GRR statistic is preferred; however, if a model-based inference is the intended goal, such as using longitudinal statistical models, the GCR is a more informative reliability statistic.^27^ In addition to the longitudinal reliability indices, we also examine factors contributing to variability both at the learner level and the program level, which inform the internal structure of the Milestones assessment system.

#### Identifying Learner Trajectories and Potential Points of Remediation

To identify different growth patterns of learners, we use longitudinal models (growth mixture models [GMMs]) to identify the shape, inflection points, and types of learning trajectories that represent subgroups of growth patterns.^28,29^ We use the quadratic GMMs to account for nonlinear growth in learners over time and also fit a traditional growth curve model for comparison.^28,29^ We reviewed inflection points in the learning curves to identify areas for early remediation for learners that could be meaningful for programs to consider.^30^ Learners with growth trajectories that do not meet the level 4 graduation target were reviewed to identify potential points of early remediation for struggling learners.

### Data Sources

We extracted data for the 6 reporting periods (2 reporting periods for each year of the 3-year family medicine residency training period) from the ACGME Accreditation Data System. We then removed all identifying learner- and program-level information prior to analysis.

### Bias

This study includes national data belonging to family medicine residents from entry to graduation who began training in 2016 and graduated in 2019. As such, we include all learners from the national database who trained in family medicine during this period, resolving potential sampling or inferential bias issues in the results.

### Study Size

Following sample size guidance for reliable and consistent estimation of GMMs that require a minimum sample size of 500 participants, our data sources provide robust sample size to make inferences regarding learning trajectories. Moreover, we checked for statistical convergence in estimates as well as statistical model fit and identification to ensure robust and consistent findings in our results.^30^

### Statistical Analysis

We use descriptive statistics and box plots to examine overall data trends in the 6 ACGME competencies and 22 family medicine subcompetencies. Reliability metrics (GRR and GCR) were estimated following the specification in Willett^11^ and in Hertzog et al.^13^ Prior studies of Milestones-based data have informed the utility of specifying program-level clustering effects on standard errors of estimates, as program variance accounts for a significant proportion of variability in the data. As such, we included program-level variance estimates into the reliability estimation and also in subsequent aspects of our analysis.^27,28^ For the GCR, we modified the model-based calculation to incorporate program-level effects.^14,26^

Growth curve models were fit for each subcompetency using the unconditional quadratic latent growth curve approach adjusted for clustering in programs.^31,32,33^ For the GMMs, we fit as many as 10 learning trajectories and used model fit indices (information criteria and classification indices) to select the best-fitting models. We reviewed plots of learning trajectories to identify potential areas for remediation based on inflection points and convergence with other growth curves within each subcompetency.^34^

Data compilation and analyses were conducted using Stata version 16 (StataCorp). We used α = .05 with 2-tailed tests to make statistical inferences. GMMs were fit using Latent Gold version 5.1 (Statistical Innovations).

## Results

### Descriptive Statistics

**Figure 1.**
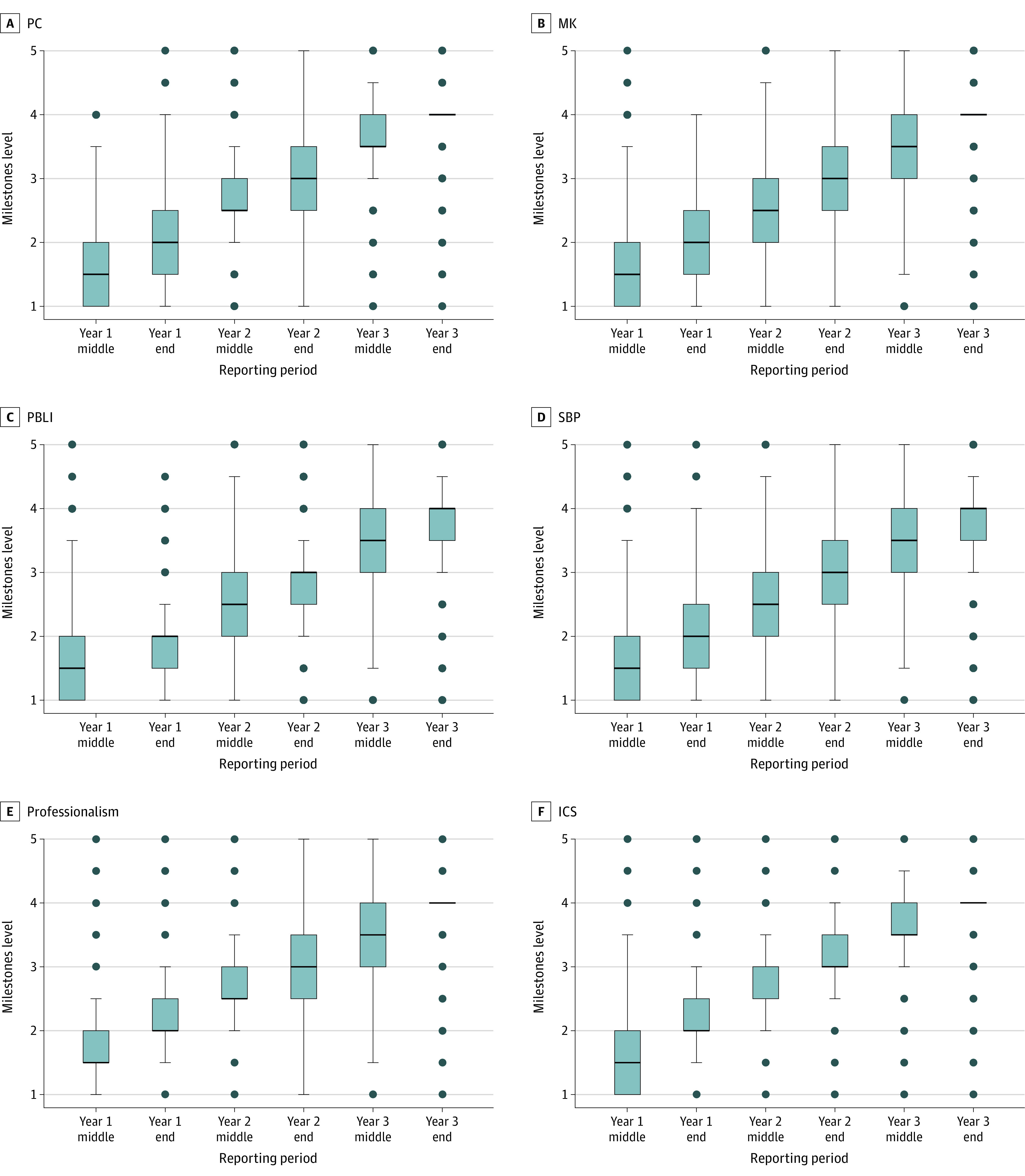
Milestones Levels by Core Competency: Box Plots by Reporting Period for 3872 Residents in 514 Programs Box plots use data for all subcompetencies within the Core Competency. The median is denoted by the line within the box; 25th percentile, bottom border of box; 75th percentile, top border of box; variability outside the IQR, whiskers; and outside values, dots. ICS indicates interpersonal and communication skills; MK, medical knowledge; PBLI, practice-based learning and improvement; PC, patient care; and SBP, system-based practice.

Milestone ratings of 3872 learners in 514 residency programs increased significantly (longitudinal mixed-effects regression) from time of entry (July 2016) to graduation (June 2019) (mean [SD] of 0.55 [0.04] Milestones units per reporting period; *P* < .001). Within this cohort, 376 residents (10%) did not graduate from their respective family medicine programs by June 2019. During the first reporting period (July to December 2016), 78 099 Milestones ratings (89%) ranged between level 1 and level 2 across all subcompetencies; at the time of graduation, 2865 learners (74%) reached level 4 (ready for unsupervised practice) or higher for all subcompetencies, leaving 1007 learners (26%) not achieving the level 4 criteria for at least 1 subcompetency. In particular, 1897 learners (49%) did not meet the level 4 criteria for PBLI-3 (improves systems in which physicians provide care); 1394 learners (36%) for SBP-2 (emphasizes patient safety), 1200 learners (31%) for professionalism-2 (demonstrates professional conduct and accountability), and 890 learners (23%) for ICS-4 (utilizes technology to optimize communication). Figure 1 illustrates the longitudinal trends in Milestones ratings using box plots across the reporting periods by ACGME core competency. For PBLI, the midyear PGY 1 median (IQR) was 1.50 (1.00-2.00), while end-of-year PGY 3 median (IQR) was 4.00 (3.50-4.00). eFigure 1 in the Supplement presents box plots for each subcompetency.

The mean baseline (ie, during the first 6 months of training) Milestones level across all subcompetencies was 1.58 (95% CI, 1.54-1.62). Subcompetencies in PC (slope, 0.57; 95% CI, 0.56-0.58), ICS (slope, 0.57; 95% CI, 0.56-0.57), and MK (slope, 0.55; 95% CI, 0.54-0.56) had the highest rates of increase over time (all *P* < .001). For example, the mean (SD) Milestones in PC increased by 0.57 (0.03) units per reporting period (in the 5-level Milestones rating scale). Details of longitudinal trends and subcompetency-level growth curves, including regression coefficients and parameter estimates, are available in the eTable in the Supplement.

### Longitudinal Reliability and Variability at Learner- and Program-Levels

#### Longitudinal Reliability

The longitudinal reliability results based on the GRR show reasonable ability to differentiate individual learner differences in slopes using the Milestones assessment system (overall mean [SD] GRR, 0.63 [0.03]). For the GCR representing model-based longitudinal reliability, the overall mean (SD) estimate was 0.91 (0.02), providing evidence that the model-based individual rates of change (ie, slopes) are precise. Table 1 shows the reliability estimates by subcompetency.

#### Program-Level Variance

There was significant program-level variability. The mean (SD) program-level variability (random-effects parameters) for Milestones ratings at baseline (July to December 2016) varied as much as 0.80 (0.08) units, depending on the program of the learner, accounting for 35% of total variance; this also applied to the rate of change, which varied by a mean (SD) of 0.18 (0.02) units or 8% of total variance depending on the program of the learner.^20^ Variability at the program level was highest for professionalism and ICS, with variability at baseline reporting 0.96 units or 38% of total variance for professionalism depending on the program and variability for change over time and 0.20 units or 9% of total variance depending on the program for ICS. Over time, program-level variance decreased for all subcompetencies except for MK-1, SBP-2, and professionalism-2, which had modest 3% increase in variability across the reporting periods. Program-level variance decreased most notably over time for PC-3, MK-2, PBLI-2, SBP-1, professionalism-3, and ICS-1, which had as much as a 16% reduction in program-level variance (eTable in the Supplement). Variability at the program level was consistently greater than at the learner level, including the rate of growth.

#### Learner-Level Variance

There was significant individual variation above and beyond program variation; however, they were substantially lower than program-level effects. Variation in individual rates of change per reporting period (accounting for 4% of total variance) was approximately half of program-level effects (accounting for 8% of total variance). Results also showed greater variability in individual- and program-level results at earlier phases of training, with greater stability in data toward later reporting periods, as noted in the negative covariance random-effects parameters (mean correlation, –0.23; 95% CI, –0.21 to –0.25). Table 1 summarizes variability at the learner and program levels, representing these effects for each subcompetency.

### Learning Trajectories

All subcompetencies generated significantly different learning trajectories of learners. Table 2 shows the number and percentage of learners assigned to different learning trajectory groups by subcompetency. In particular, 8 subcompetencies (PC-2, PC-3, PC-4, PC-5, MK-1, SBP-3, SBP-4, and professionalism-3) had 3 learning trajectory groups of growth patterns per learner; the remaining 14 subcompetencies (PC-1, MK-2, PBLI-1, PBLI-2, PBLI-3, SBP-1, SBP-2, professionalism-1, professionalism-2, professionalism-4, ICS-1, ICS-2, ICS-3, and ICS-4) had 4 learning trajectory groups. The distribution of learners classified to each group ranged significantly, reflecting different patterns of developmental growth (eFigures 2-7 in the Supplement).

**Table 2.** Number and Percentage of Learners Assigned to Different Learning Trajectory Groups by Subcompetency

ACGME core competency and subcompetencies	Learning trajectory groups, No. (%)^a^			
	1	2	3	4
PC				
PC-1	1007 (26)	542 (14)	1316 (34)	1007 (26)
PC-2	929 (24)	1781 (46)	1162 (30)	NA
PC-3	1704 (44)	929 (24)	1239 (32)	NA
PC-4	1007 (26)	1742 (45)	1123 (29)	NA
PC-5	1394 (36)	891 (23)	1587 (41)	NA
MK				
MK-1	774 (20)	2091 (54)	1007 (26)	NA
MK-2	1084 (28)	465 (12)	1510 (39)	813 (21)
SBP				
SBP-1	1123 (29)	503 (13)	851 (22)	1355 (35)
SBP-2	542 (14)	1278 (33)	813 (21)^b^	1239 (32)
SBP-3	1665 (43)	891 (23)	1316 (34)	NA
SBP-4	1355 (35)	813 (21)	1704 (44)	NA
PBLI				
PBLI-1	658 (17)	1355 (35)	1123 (29)^b^	736 (19)^b^
PBLI-2	736 (19)	1354 (35)	620 (16)^b^	1162 (30)
PBLI-3	697 (18)	1394 (36)	929 (24)^b^	852 (22)
Professionalism				
Professionalism-1	736 (19)	1277 (33)	581 (15)^b^	1278 (33)
Professionalism-2	696 (18)	1394 (36)	620 (16)^b^	1162 (30)
Professionalism-3	929 (24)	1936 (50)	1007 (26)^b^	NA
Professionalism-4	852 (22)	1316 (34)	581 (15)^b^	1123 (29)
ICS				
ICS-1	620 (16)	1123 (29)	929 (24)	1200 (31)
ICS-2	735 (19)	1394 (36)	581 (15)^b^	1162 (30)
ICS-3	774 (20)	1355 (35)	620 (16)^b^	1123 (29)
ICS-4	852 (22)	1665 (43)	503 (13)^b^	852 (22)

Abbreviations: ACGME, Accreditation Council for Graduate Medical Education; ICS, interpersonal and communication skills; MK, medical knowledge; NA, not applicable; PC, patient care; PBLI, practice-based learning and improvement; SBP, system-based practice.

^a^ Learners in group 1 had the highest Milestones rating at baseline (first reporting period), followed by learners in groups 2, 3, and 4, respectively. Some subcompetencies do not have a fourth group. See eFigure 2 to eFigure 7 in the Supplement for illustrations of learning trajectories specific to each group by subcompetency.

^b^ Latent trajectory subgroups that did not meet graduation target of level 4.

In Table 2, there were 11 subcompetencies (SBP-2, PBLI-1, PBLI-2, PBLI-3, professionalism-1, professionalism-2, professionalism-3, professionalism-4, ICS-2, ICS-3, and ICS4) with learning trajectory subgroups that would not reach the level 4 target; the percentage of learners that fit into this learning trajectory ranged from 13% to 29%, representing a significant proportion of residents. For example, in SBP-2, 813 learners (21%) classified to learning trajectory group 3 were not estimated to reach the level 4 graduation target.

### Identifying Potential Points of Remediation

**Figure 2.**
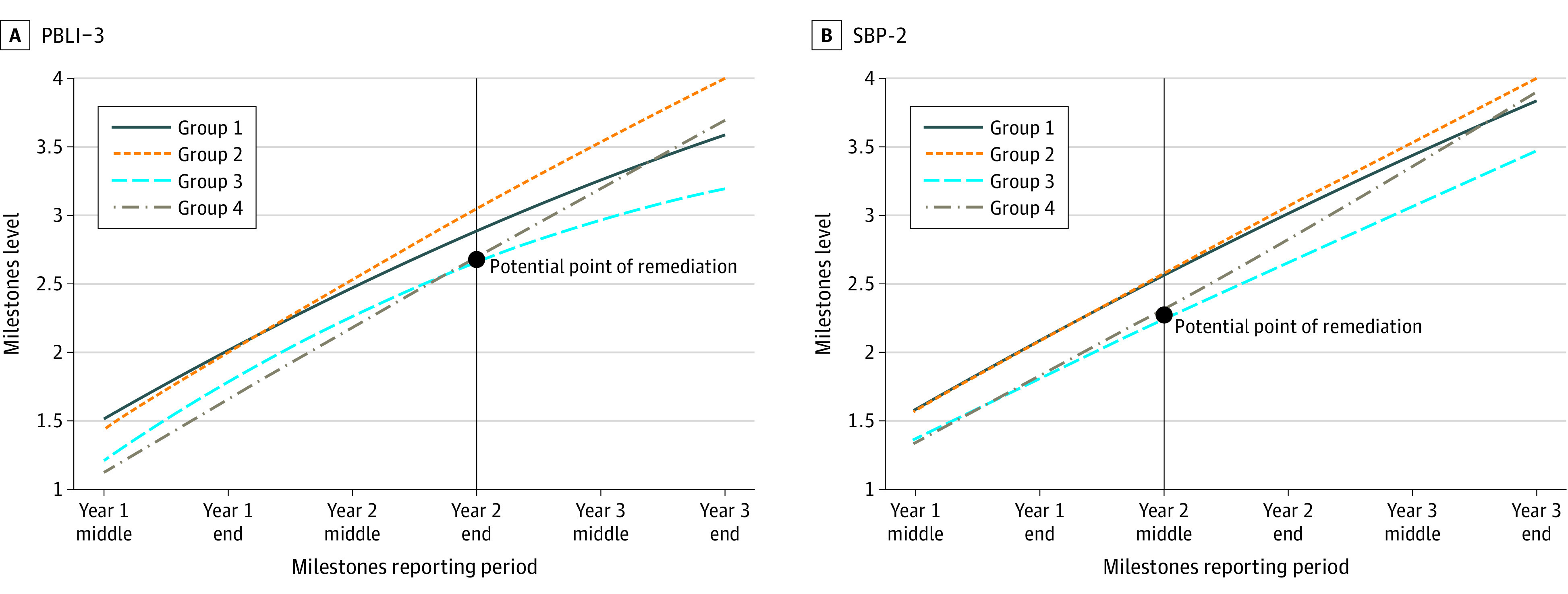
Growth Trajectories for Practice-Based Learning and Improvement–3 (PBLI-3) and Systems-Based Practice–2 (SBP-2) PBLI-3 is improves systems in which the physician provides care; SBP-2, emphasizes patient safety. Learning trajectories reflect uniquely distinct pathways of learners, as identified from the national data. Learners in group 3 did not achieve the level 4 graduation target indicating that they are ready for unsupervised practice.

Figure 2 shows learning trajectories for PBLI-3 and SBP-2 . Both panels have 4 learning trajectories: groups 1 and 2 have higher Milestones ratings at baseline (PGY 1 midyear reporting period) relative to groups 3 and 4. For PBLI-3, both group 3 (929 residents [24%]) and group 4 (852 residents [22%]) have similar longitudinal progress between midyear PGY 1 to end-year PGY 2 end-year reporting periods; however, their trajectories diverged at the end of PGY 2, with group 3 learners’ developmental progress plateauing and not meeting the graduation target. We saw a similar pattern for SBP-2, where residents in group 3 (813 residents [21%]) and group 4 (1239 residents [32%]) had similar growth trajectories from midyear PGY 1 to midyear PGY 2, but their trajectories began to diverge from the midyear PGY 2 midyear reporting period. Residents in group 3 were not estimated to meet the graduation target. In both figures, groups 1, 2, and 4 ultimately met the graduation target, but learners in group 3 diverged and their performance began to plateau.

**Figure 3.**
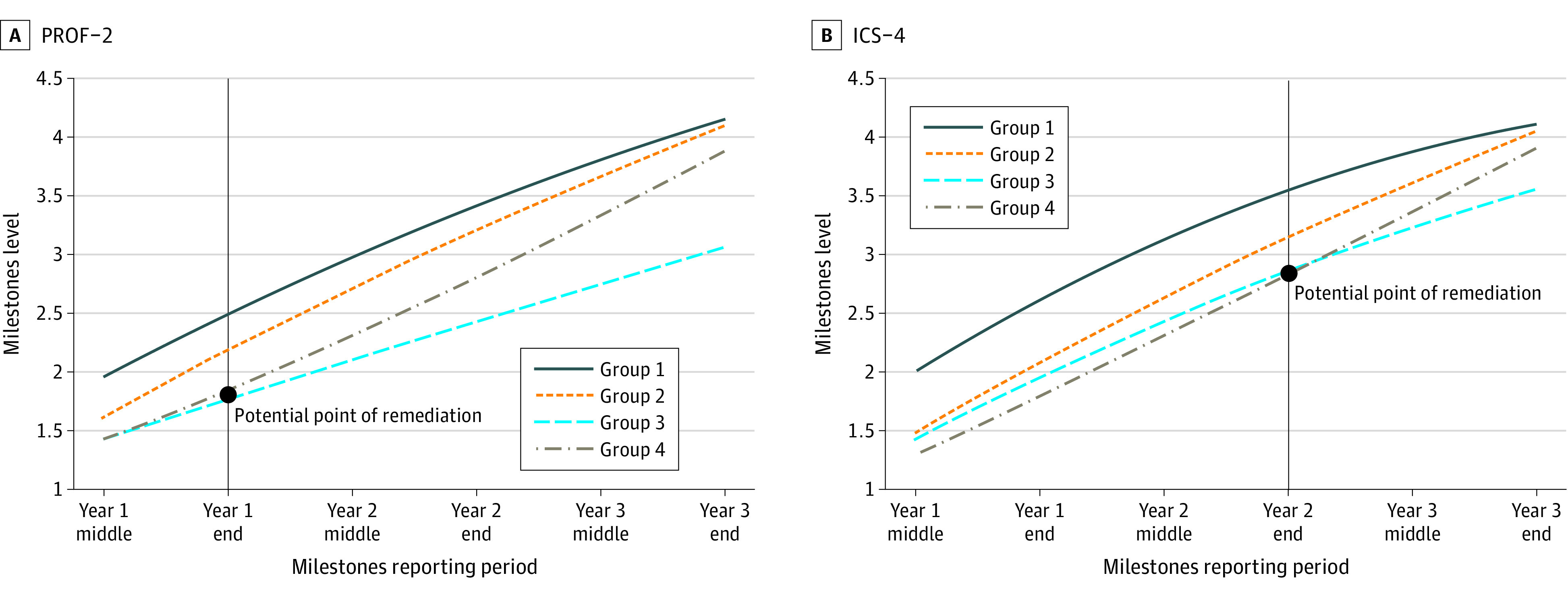
Growth Trajectories for Professionalism-2 (PROF-2) and Interpersonal and Communication Skills–4 (ICS-4) PROF-2 is demonstrates professional conduct and accountability; ICS-4, utilizes technology to optimize communication. Learning trajectories reflect uniquely distinct pathways of learners, as identified from the national data. Learners in group 3 did not achieve the level 4 graduation target indicating ready for unsupervised practice.

Figure 3 shows a similar illustration of learning trajectories for professionalism-2 and ICS-4. In both cases, learners in group 3 (professionalism-2, 620 residents [16%]; ICS-4, 503 residents [13%]) had trajectories that plateaued and did not meet the graduation target.

## Discussion

Residency is an intensely developmental experience as the new physician begins their journey in becoming a specialty physician. Competency frameworks in education include a longitudinal component by design, prompting educators to examine how learners develop and meet thresholds for competence.^1,4,18^ This study used national data to examine longitudinal consistency and reproducibility of the Milestones assessment system, leveraging a continuum of developmental learning progression studies from the microlevel (individual learner growth) to the macrolevel (population growth curves). This work focuses on a meso-level (ie, patterns of growth shared by groups of learners). Identifying different patterns of learning trajectories can serve to target and remediate learners who may show signs of difficulty in their training. Identifying learning trajectories could also allow researchers to study factors that may mediate the learning progress.^19^

Using national data, this study showed varying developmental trajectories of residents, with significant differences in their growth patterns that can be used for educational improvement and programmatic uses. Growth curves and learning analytics are important tools that have the potential to allow program directors and trainees to follow trajectories of competency acquisition, thereby allowing for early identification of struggling, average, and exceptional residents. For learners, these tools allow them to use such information to inform their individualized learning plans. As such, identifying each type of trainee could allow for further individualization of training for all learners, not just those who are struggling.^4,35,36,37,38^ Findings from this study suggest that the Milestones reporting assessment system provides reliable longitudinal assessment data for monitoring individualized developmental progress of learners across all subcompetencies, supporting the use of Milestones data to inform and guide individualized learning.^17^

We found greater variation at the learner and program level in earlier phases in training, which may be because of a combination of actual learner plateaus or ceiling effects in the Milestones.^17^ In particular, we found significant differences in learners classified to different growth trajectories, particularly relating to competencies in PBLI, SBP, professionalism, and ICS. As examples, we presented detailed illustrations (Figure 2 and Figure 3). There may be several explanations related to distinct growth trajectories in PBLI-3 (improves systems in which the physician provides care), professionalism-2 (demonstrates professional conduct and accountability), ICS-4 (utilizes technology to optimize communication), and SBP-2 (emphasizes patient safety); they may be because of challenges in teaching and assessment characteristics in each of these subcompetencies. These training periods, when resident performance begins to slow, can be educationally meaningful time points for remediation and can be informative for both the learner and the program director. The time points where trajectories diverge can be marked as potential points of remediation for these subcompetencies. Additional work should also examine the educational setting and learning environments used for measuring these subcompetencies for determining the Milestones levels.

An important consideration when conducting Milestones-based data analysis is to incorporate program-level effects, as program variance accounted for a greater proportion of overall variance than beyond individual effects, consistent with earlier study of pediatrics milestones by Hu and colleagues.^20,39^ This study showed that program-level variance is more than 60% greater in proportion than learner-level variance, indicating substantial variability due to programs. On average, learner variance accounted for 22% of total variance, while program-level variance contributed to 35% of variance, with varying changes in program-level variance by subcompetency over time. In addition, variability in longitudinal growth at the program level was nearly double the proportion of learners, indicating that programs are responsible for the degree of developmental progress of learners. Beyond program-level variance, longitudinal variability is also largely driven by programs; that is, the rate of growth (ie, slope) and the inflection points associated with learning trajectories are more heavily associated with the program than the individual learner. Future studies should explore the degree to which learning trajectories are affected by program-level straight-line scoring (learners receiving the same Milestones rating across subcompetencies), leniency or stringency factors, and other response process issues at the CCC as sources of construct irrelevant variance.^17,20,37,39^

Learning trajectories with supporting reliability evidence as identified in this study can be used to develop and inform individualized learning plans and remediation.^19^ These findings are consistent with prior evidence that support using the Milestones assessment system to make inferences and to build learner analytics systems that can inform resident progress and learning.^20^ Across all subcompetencies, there were 3 or 4 groups of learning trajectories that varied by shape and inflection points. The shapes of trajectories could indicate meaningful time points for remediation (ranging from PGY 1 and PGY 2 periods) that can help learners meet level 4 graduation targets. Learning trajectories in PC and MK subcompetencies tended to all reach the level 4 subcompetency at a similar rate, possibly because of greater attention on these competencies within programs; greater variation and divergence were observed for PBLI, SBP, professionalism, and ICS, prompting attention in these areas.

### Limitations

This study has limitations. Additional studies in the future can inform differences in learner pathways for residents who had difficulty completing training (and did not graduate) or who switched programs. This study found that approximately 10% of learners did not graduate in family medicine within the 3-year training period, warranting further study of the reasons why residents did not graduate in time and which residents did not meet graduation targets.^40^ As noted in prior studies,^20^ CCCs have variation in how they interpret and synthesize learner assessment data and may include rating severity and/or leniency errors over time, which this study cannot directly control, affecting the response process validity issues in the data. CCCs may also have varying frames of references for longitudinal Milestones judgments, including their own growth standards, potentially affecting the developmental pathway of individual learners and residents within the program. Additional work is also needed to examine response process issues, including factors that may pose potential bias in Milestone assessment ratings because of gender, race, and ethnicity. To the extent possible, we incorporated program-level variability in our analyses to account for these differences; nevertheless, additional consideration on the quality of data reported should continue to be investigated in future work. Additionally, this study did not explore reasons why certain learners did not meet level 4 graduation targets or how a potential remediation may be beneficial in mitigating the performance of struggling learners. Aligning these findings with the emerging data on Milestones 2.0 remains an area for future work.^41^

## Conclusions

This study found that the internal structure of Milestones data, targeting the longitudinal consistency and reliability, were valid, providing confidence to make individual growth predictions and to use the Milestones-based data to make formative developmental decisions and offer feedback to learners. We found considerable variability at the program level, which needs to be considered when conducting large-scale inferences. Moreover, learners progressed in significantly different patterns depending on the subcompetency. Programs should consider using the Milestones data to identify areas for early remediation and to differentiate learning pathways depending on the subcompetencies targeted.

## Notes

Supplement. **eTable.** Longitudinal Analysis: Quadratic Growth Curve Analysis for 3872 Learners in 514 Programs **eFigure 1.** National Level Milestone Ratings by Subcompetency: Box Plots by Reporting Period for 3872 Learners in 514 Programs **eFigure 2.** Growth Curve Trajectories for Family Medicine Subcompetencies: Practice-Based Learning and Improvement (PBLI) **eFigure 3.** Growth Curve Trajectories for Family Medicine Subcompetencies: Systems-Based Practice (SBP) **eFigure 4.** Growth Curve Trajectories for Family Medicine Subcompetencies: Professionalism **eFigure 5.** Growth Curve Trajectories for Family Medicine Subcompetencies: Interpersonal Communication Skills (ICS) **eFigure 6.** Growth Curve Trajectories for Family Medicine Subcompetencies: Patient Care (PC) **eFigure 7.** Growth Curve Trajectories for Family Medicine Subcompetencies: Medical Knowledge (MK)
