# Supplementary material for: Longitudinal Reliability of Milestones-Based Learning Trajectories in Family Medicine Residents
Source: JAMA Netw Open. 2021 Dec 7;4(12):e2137179. doi: 10.1001/jamanetworkopen.2021.37179 (PMC8652607; doi:10.1001/jamanetworkopen.2021.37179)
Supplement: Supplement. — eTable. Longitudinal Analysis: Quadratic Growth Curve Analysis for 3872 Learners in 514 Programs eFigure 1. National Level Milestone Ratings by Subcompetency: Box Plots by Reporting Period for 3872 Learners in 514 Programs eFigure 2. Growth Curve Trajectories for Family Medicine Subcompetencies: Practice-Based Learning and Improvement (PBLI) eFigure 3. Growth Curve Trajectories for Family Medicine Subcompetencies: Systems-Based Practice (SBP) eFigure 4. Growth Curve Trajectories for Family Medicine Subcompetencies: Professionalism eFigure 5. Growth Curve Trajectories for Family Medicine Subcompetencies: Interpersonal Communication Skills (ICS) eFigure 6. Growth Curve Trajectories for Family Medicine Subcompetencies: Patient Care (PC) eFigure 7. Growth Curve Trajectories for Family Medicine Subcompetencies: Medical Knowledge (MK) [file jamanetwopen-e2137179-s001.pdf]

Supplemental Online Content

Park YS, Hamstra SJ, Yamazaki K, Holmboe E. Longitudinal reliability of Milestones-based learning trajectories in family medicine residents. *JAMA Netw Open.* 2021;4(12):e2137179. doi:10.1001/jamanetworkopen.2021.37179

eTable. Longitudinal Analysis: Quadratic Growth Curve Analysis for 3872 Learners in 514 Programs

eFigure 1. National Level Milestone Ratings by Subcompetency: Box Plots by Reporting Period for 3872 Learners in 514 Programs

eFigure 2. Growth Curve Trajectories for Family Medicine Subcompetencies: Practice-Based Learning and Improvement (PBLI)

eFigure 3. Growth Curve Trajectories for Family Medicine Subcompetencies: Systems-Based Practice (SBP)

eFigure 4. Growth Curve Trajectories for Family Medicine Subcompetencies: Professionalism

eFigure 5. Growth Curve Trajectories for Family Medicine Subcompetencies: Interpersonal Communication Skills (ICS)

eFigure 6. Growth Curve Trajectories for Family Medicine Subcompetencies: Patient Care (PC)

eFigure 7. Growth Curve Trajectories for Family Medicine Subcompetencies: Medical Knowledge (MK)

This supplemental material has been provided by the authors to give readers additional information about their work.

© 2021 Park YS et al. *JAMA Network Open.*

eTable. Longitudinal Analysis: Quadratic Growth Curve Analysis for 3872 Learners in 514 Programs

ACGME Core Competency	Family Medicine Subcompetency[a]	Fixed-Effect Estimates[b,c]						Random-Effect Estimates[b,c]											
								Program-Level Estimates				Learner-Level Estimates						SD(residual)	
		Linear Time		Quadratic Time		Intercept		SD(Time)		SD(Intercept)		SD(Time)		SD(Intercept)		*r* (Time, Intercept)			
Patient Care	PC-1	.61	(.01)	–.02	(.00)	1.53	(.02)	.08	(.00)	.38	(.01)	.04	(.00)	.28	(.01)	–.37	(.04)	.35	(.00)
	PC-2	.57	(.01)	–.01	(.00)	1.54	(.02)	.08	(.00)	.36	(.01)	.04	(.00)	.23	(.01)	–.17	(.06)	.33	(.00)
	PC-3	.55	(.01)	–.02	(.00)	1.56	(.02)	.08	(.00)	.37	(.01)	.04	(.00)	.23	(.01)	–.48	(.04)	.32	(.00)
	PC-4	.60	(.01)	–.02	(.00)	1.45	(.02)	.08	(.00)	.37	(.01)	.05	(.00)	.26	(.01)	–.33	(.05)	.35	(.00)
	PC-5	.54	(.01)	–.01	(.00)	1.48	(.02)	.08	(.00)	.36	(.01)	.05	(.00)	.25	(.01)	–.29	(.05)	.36	(.00)
Medical Knowledge	MK-1	.54	(.01)	–.01	(.00)	1.54	(.02)	.08	(.00)	.36	(.01)	.02	(.01)	.27	(.01)	.11	(.17)	.42	(.00)
	MK-2	.56	(.01)	–.02	(.00)	1.54	(.02)	.07	(.00)	.36	(.01)	.05	(.00)	.29	(.01)	–.43	(.04)	.34	(.00)
Practice-Based Learning and Improvement	PBLI-1	.51	(.01)	.00	(.00)	1.41	(.02)	.10	(.00)	.40	(.01)	.04	(.00)	.23	(.01)	–.08	(.08)	.36	(.00)
	PBLI-2	.53	(.01)	–.01	(.00)	1.64	(.02)	.08	(.00)	.39	(.01)	.05	(.00)	.28	(.01)	–.42	(.04)	.36	(.00)
	PBLI-3	.53	(.01)	–.02	(.00)	1.36	(.02)	.10	(.00)	.39	(.01)	.04	(.00)	.18	(.01)	–.07	(.09)	.37	(.00)
System-Based Practice	SBP-1	.60	(.01)	–.02	(.00)	1.46	(.02)	.08	(.00)	.39	(.01)	.04	(.00)	.23	(.01)	–.46	(.05)	.34	(.00)
	SBP-2	.47	(.01)	.00	(.00)	1.49	(.02)	.09	(.00)	.35	(.01)	.04	(.00)	.18	(.01)	.13	(.09)	.34	(.00)
	SBP-3	.51	(.01)	–.01	(.00)	1.57	(.02)	.10	(.00)	.40	(.01)	.04	(.00)	.19	(.01)	–.07	(.07)	.34	(.00)
	SBP-4	.58	(.01)	–.02	(.00)	1.64	(.02)	.08	(.00)	.39	(.01)	.05	(.00)	.26	(.01)	–.35	(.04)	.36	(.00)
Professionalism	PROF-1	.60	(.01)	–.03	(.00)	1.62	(.02)	.10	(.00)	.49	(.02)	.06	(.00)	.28	(.01)	–.20	(.05)	.41	(.00)
	PROF-2	.47	(.01)	–.01	(.00)	1.66	(.02)	.09	(.00)	.43	(.02)	.08	(.00)	.25	(.01)	.11	(.06)	.41	(.00)
	PROF-3	.56	(.01)	–.02	(.00)	1.71	(.02)	.09	(.00)	.46	(.02)	.05	(.00)	.26	(.01)	–.49	(.04)	.36	(.00)
	PROF-4	.50	(.01)	–.01	(.00)	1.73	(.02)	.09	(.00)	.42	(.02)	.06	(.00)	.26	(.01)	–.25	(.04)	.38	(.00)
Interpersonal Communication Skills	ICS-1	.55	(.01)	–.02	(.00)	1.86	(.02)	.09	(.00)	.45	(.02)	.06	(.00)	.29	(.01)	–.47	(.03)	.35	(.00)
	ICS-2	.59	(.01)	–.02	(.00)	1.62	(.02)	.09	(.00)	.44	(.02)	.05	(.00)	.27	(.01)	–.40	(.04)	.36	(.00)
	ICS-3	.55	(.01)	–.02	(.00)	1.70	(.02)	.10	(.00)	.43	(.02)	.06	(.00)	.29	(.01)	–.36	(.04)	.36	(.00)
	ICS-4	.57	(.01)	–.02	(.00)	1.59	(.02)	.10	(.00)	.49	(.02)	.05	(.00)	.25	(.01)	–.09	(.07)	.40	(.00)

Note:

a. PC-1: Cares for acutely ill or injured patients in urgent and emergent situations and in all settings; PC-2: Cares for patients with chronic conditions; PC-3: Partners with the patient, family, and community to improve health through disease prevention and health promotion; PC-4: Partners with the patient to address issues of ongoing signs, symptoms, or health concerns that remain over time without clear diagnosis despite evaluation and treatment, in a patient-centered, cost-effective manner; PC-5: Performs specialty-appropriate procedures to meet the health care needs of individual patients, families, and communities, and is knowledgeable about procedures performed by other specialists to guide their patients' care; MK-1: Demonstrates medical knowledge of sufficient breadth and depth to practice family medicine; MK-2: Applies critical thinking skills in patient care; SBP-1: Provides cost-conscious medical care; SBP-2: Emphasizes patient safety; SBP-3: Advocates for individual and community health; SBP-4: Coordinates team-based care; PBLI -1 Locates, appraises, and assimilates evidence from scientific studies related to the patients' health problems; PBLI-2: Demonstrates self-directed learning; PBLI-3: Improves systems in which the physician provides care; PROF-1: Completes a process of professionalization; PROF-2: Demonstrates professional conduct and accountability; PROF-3: Demonstrates humanism and cultural proficiency; PROF-4: Maintains emotional, physical, and mental health; and pursues continual personal and professional growth; ICS-1: Develops meaningful, therapeutic relationships with patients and families; ICS-2: Communicates effectively with patients, families, and the public; ICS-3: Develops relationships and effectively communicates with physicians, other health professionals, and health care teams; ICS-4: Utilizes technology to optimize communication.

b. All estimates $P < .001$. Values in parenthesis are standard errors.

c. Interpretation for this table can be made as follows. For example, in PC-1 (first row of Table 2), the baseline Milestone level during the first six months of training is 1.53 (95% CI: 1.49, 1.57), with an average increase in Milestone levels by .61 units (95% CI: .59, .63) for every additional Milestone-reporting period; the significant quadratic-effect (coefficient = –.02 [SE = .00]) indicates non-linear growth. While the mean baseline Milestone level for PC-1 is estimated at 1.53 (fixed-effect

© 2021 Park YS et al. *JAMA Network Open.*

intercept), the program-level variability (standard deviation) is .38 (95% CI: .36, .40), while learner variability is .28 (95% CI: .26, .30), indicating significant variability both at the program and learner levels at baseline. In addition, the slope (rate of growth) also demonstrates significant variability (standard deviation) both at the program level, .08 (95% CI: .08, .09), and at the individual level, .04 (95% CI: .04, .05), also indicating variability in rates of growth both at the program and learner levels.

© 2021 Park YS et al. *JAMA Network Open.*

eFigure 1. National Level Milestone Ratings by Subcompetency:[a] Box Plots by Reporting Period[b] for 3872 Learners in 514 Programs

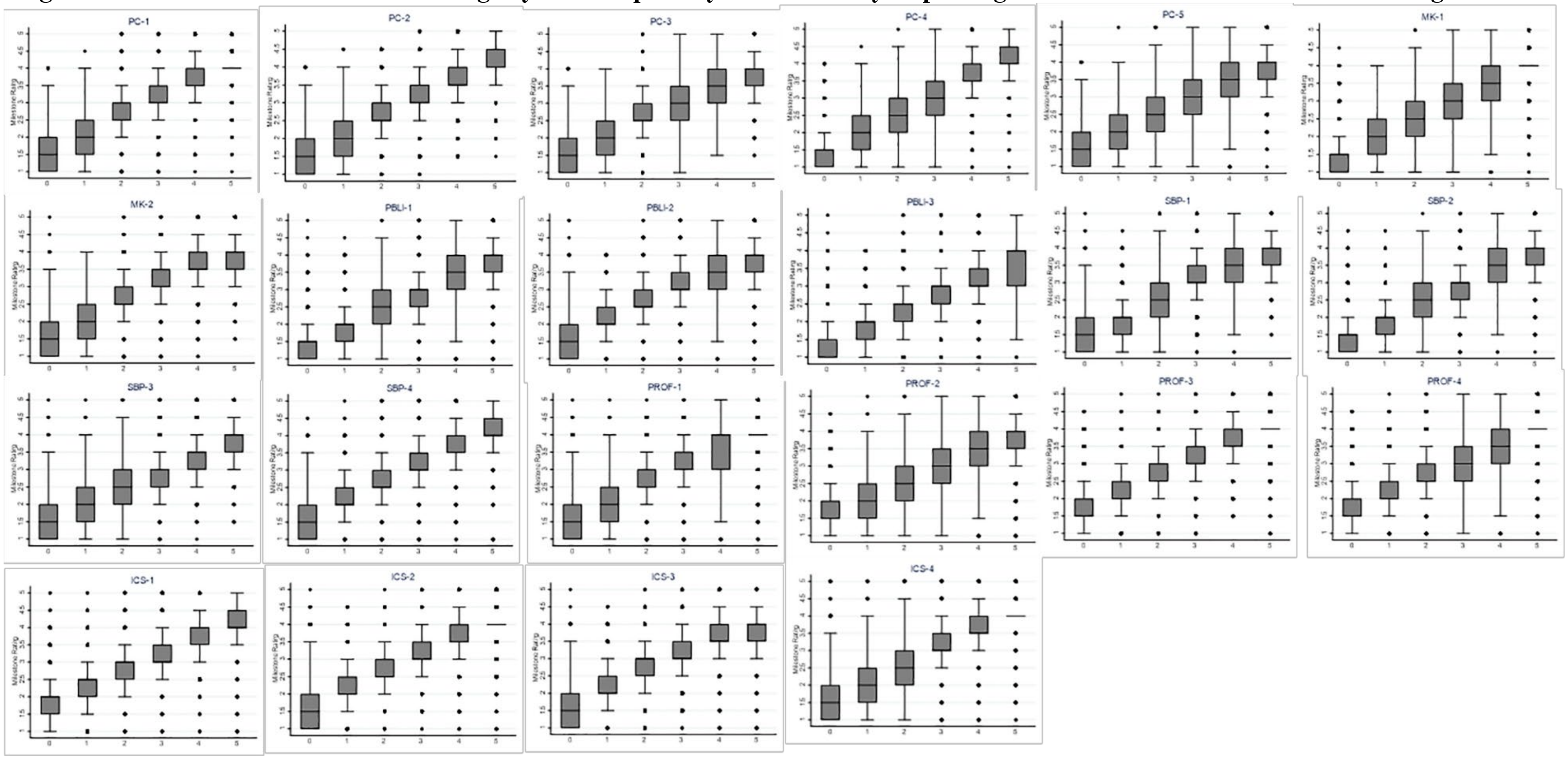

Note: a. PC-1: Cares for acutely ill or injured patients in urgent and emergent situations and in all settings; PC-2: Cares for patients with chronic conditions; PC-3: Partners with the patient, family, and community to improve health through disease prevention and health promotion; PC-4: Partners with the patient to address issues of ongoing signs, symptoms, or health concerns that remain over time without clear diagnosis despite evaluation and treatment, in a patient-centered, cost-effective manner; PC-5: Performs specialty-appropriate procedures to meet the health care needs of individual patients, families, and communities, and is knowledgeable about procedures performed by other specialists to guide their patients' care; MK-1: Demonstrates medical knowledge of sufficient breadth and depth to practice family medicine; MK-2: Applies critical thinking skills in patient care; SBP-1: Provides cost-conscious medical care; SBP-2: Emphasizes patient safety; SBP-3: Advocates for individual and community health; SBP-4: Coordinates team-based care; PBLI -1 Locates, appraises, and assimilates evidence from scientific studies related to the patients' health problems; PBLI-2: Demonstrates self-directed learning; PBLI-3: Improves systems in which the physician provides care; PROF-1: Completes a process of professionalization; PROF-2: Demonstrates professional conduct and accountability; PROF-3: Demonstrates humanism and cultural proficiency; PROF-4: Maintains emotional, physical, and mental health; and pursues continual personal and professional growth; ICS-1: Develops meaningful, therapeutic relationships with patients and families; ICS-2: Communicates effectively with patients, families, and the public; ICS-3: Develops relationships and effectively communicates with physicians, other health professionals, and health care teams; ICS-4: Utilizes technology to optimize communication; b. X-axis shows mid- and end-of-year reporting periods ("0": Y1-Mid, "1": Y1-End, "2": Y2-Mid, "3": Y2-End, "4": Y3-Mid, "5": Y3-End).

© 2021 Park YS et al. *JAMA Network Open.*

eFigure 2. Growth Curve Trajectories for Family Medicine Subcompetencies: Practice-Based Learning and Improvement (PBLI)

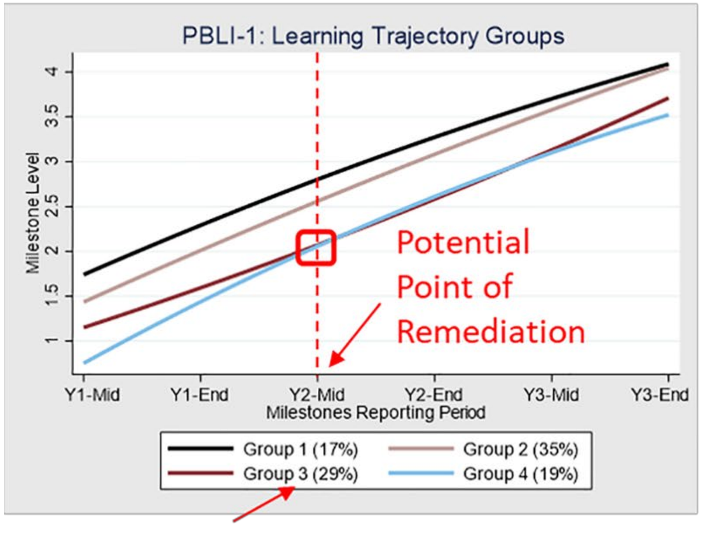

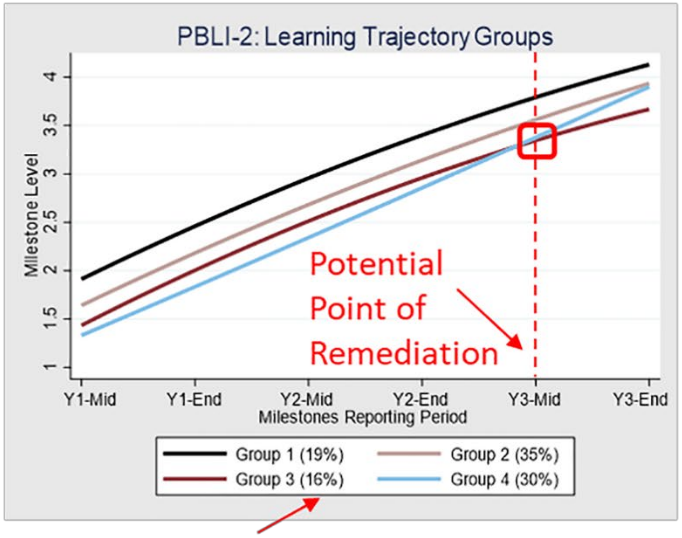

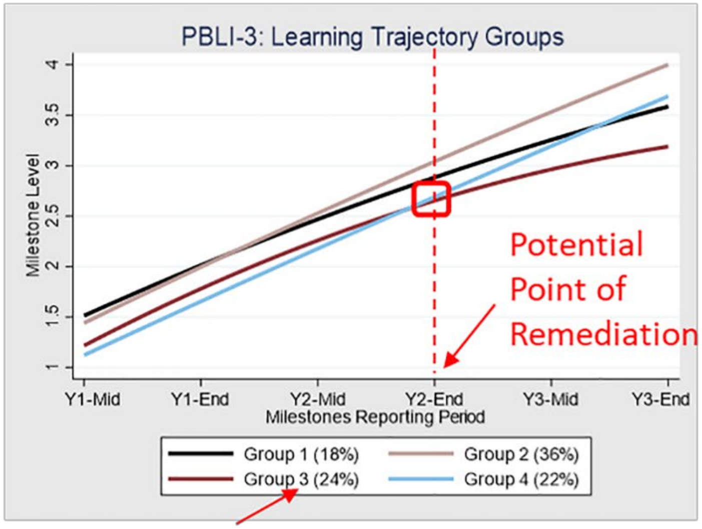

© 2021 Park YS et al. *JAMA Network Open.*

eFigure 3. Growth Curve Trajectories for Family Medicine Subcompetencies: Systems-Based Practice (SBP)

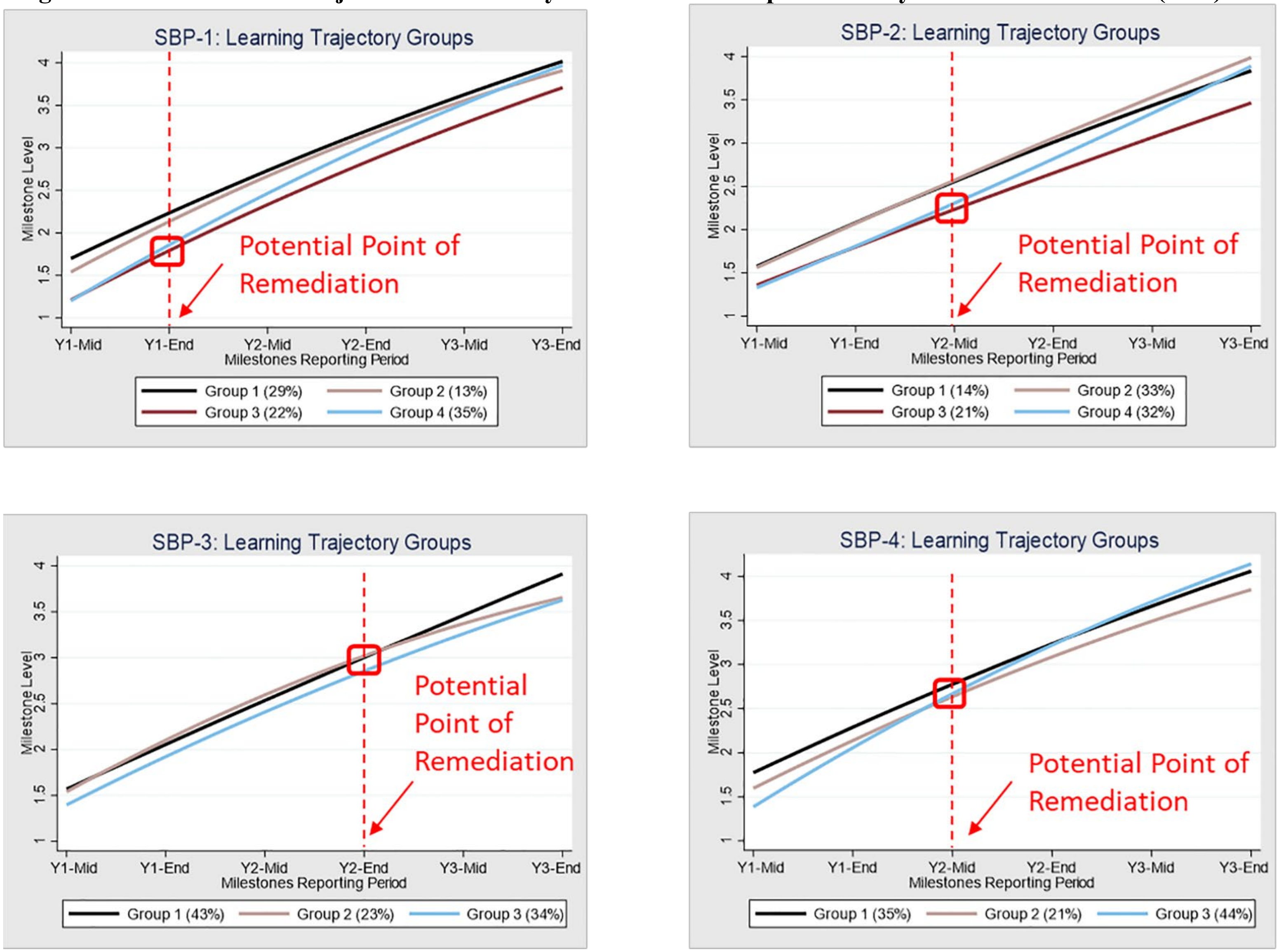

© 2021 Park YS et al. *JAMA Network Open.*

eFigure 4. Growth Curve Trajectories for Family Medicine Subcompetencies: Professionalism (PROF)

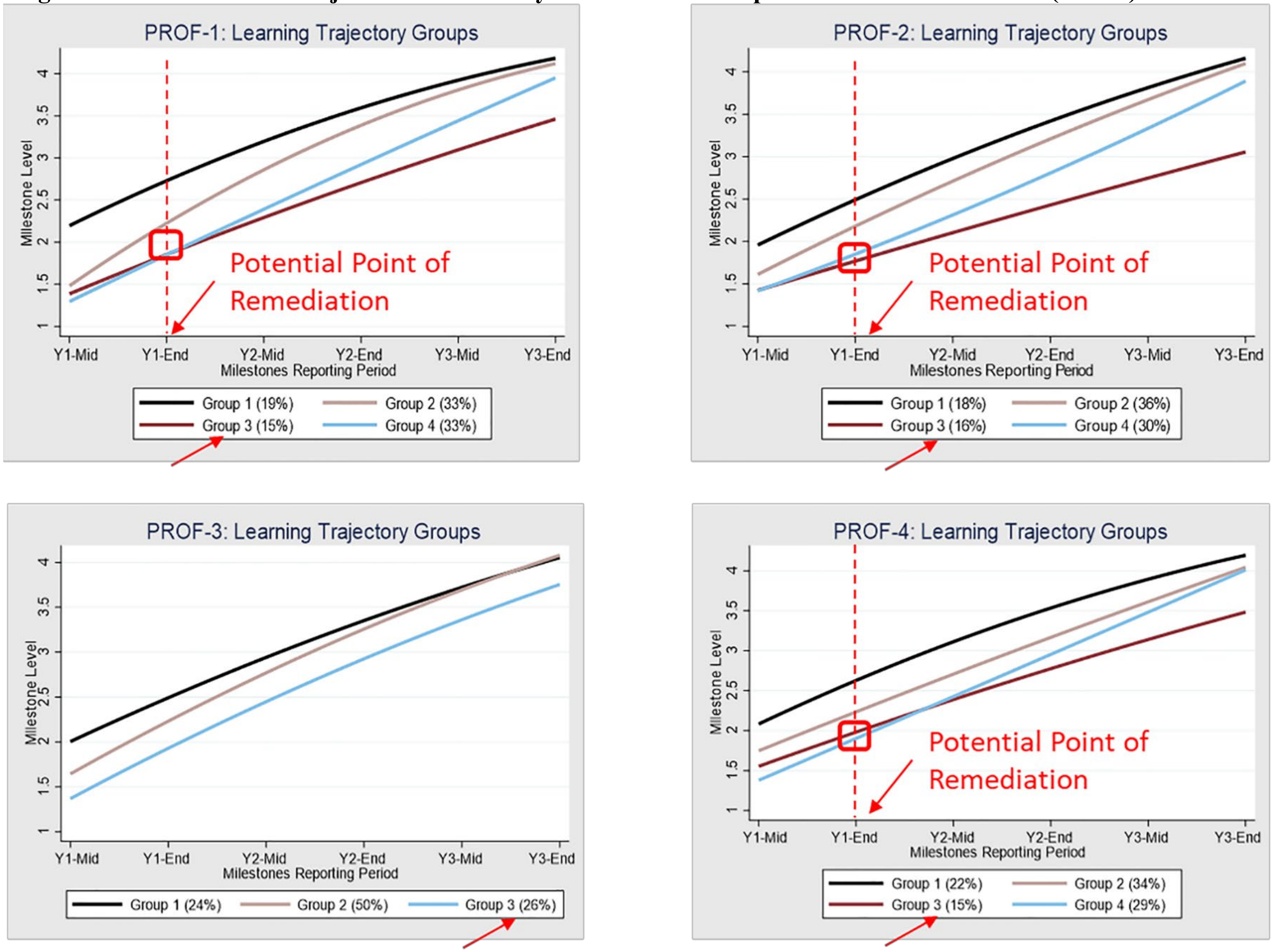

© 2021 Park YS et al. *JAMA Network Open.*

eFigure 5. Growth Curve Trajectories for Family Medicine Subcompetencies: Interpersonal Communication Skills (ICS)

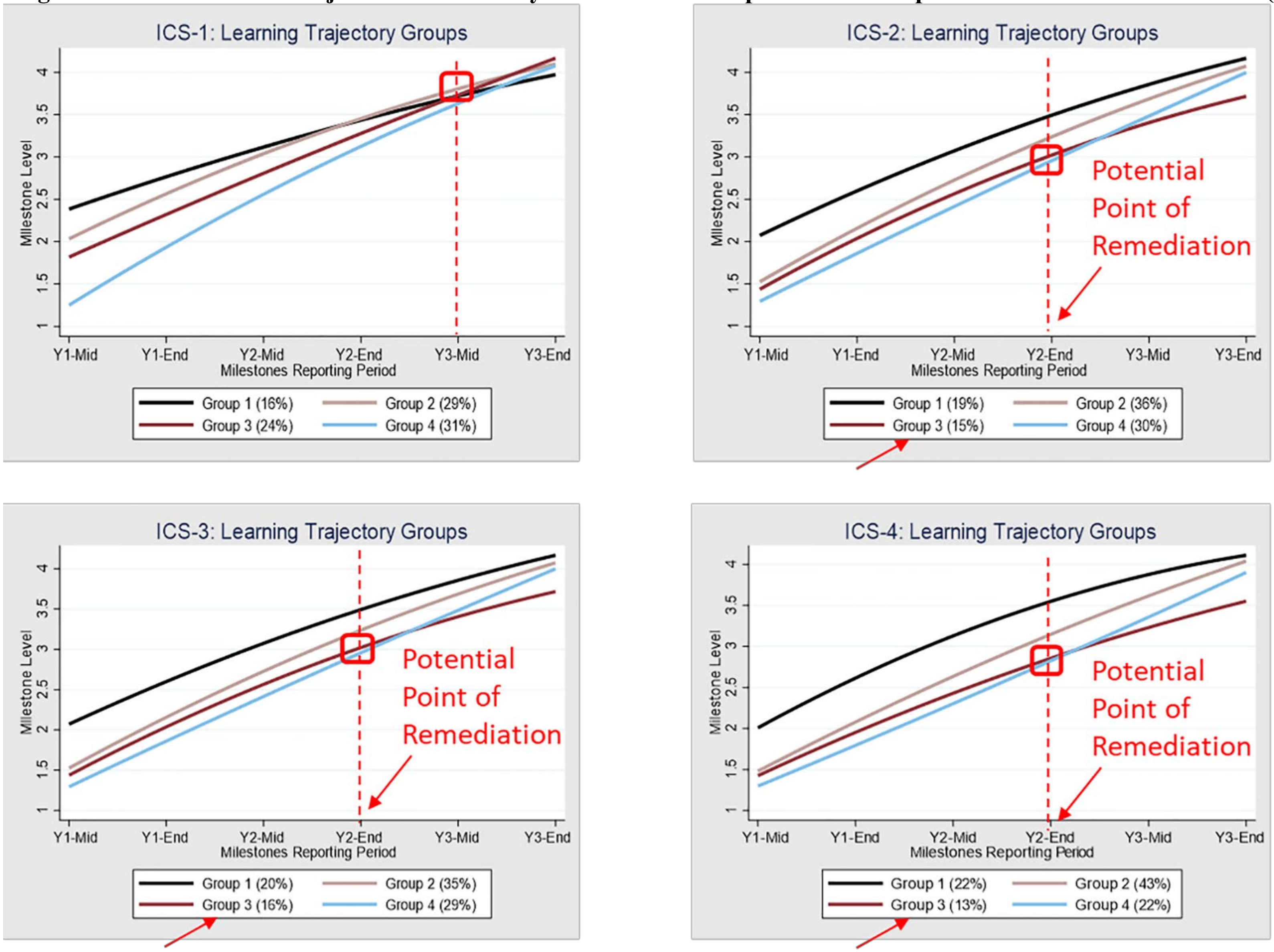

© 2021 Park YS et al. *JAMA Network Open.*

eFigure 6. Growth Curve Trajectories for Family Medicine Subcompetencies: Patient Care (PC)

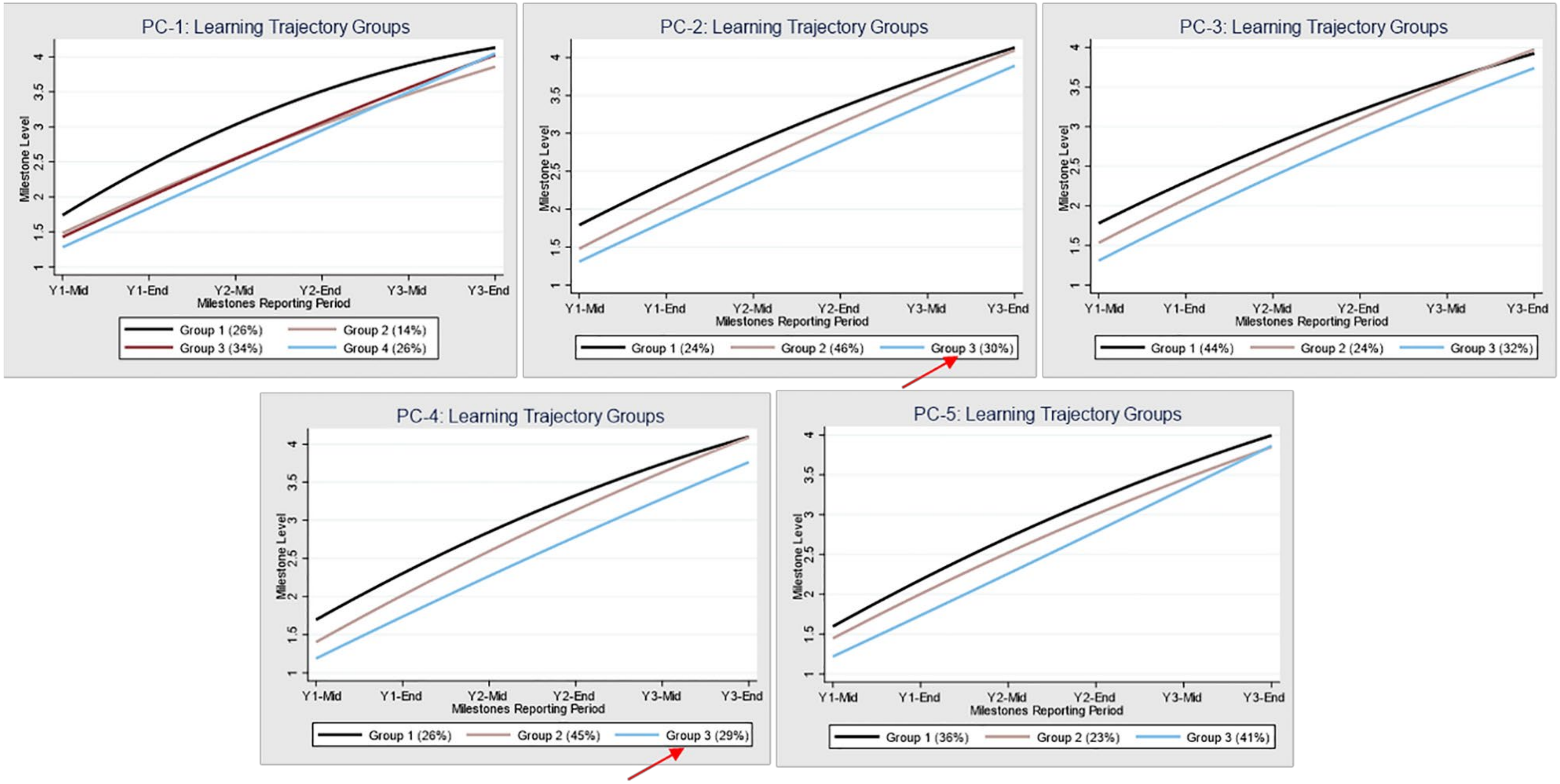

© 2021 Park YS et al. *JAMA Network Open.*

eFigure 7. Growth Curve Trajectories for Family Medicine Subcompetencies: Medical Knowledge (MK)

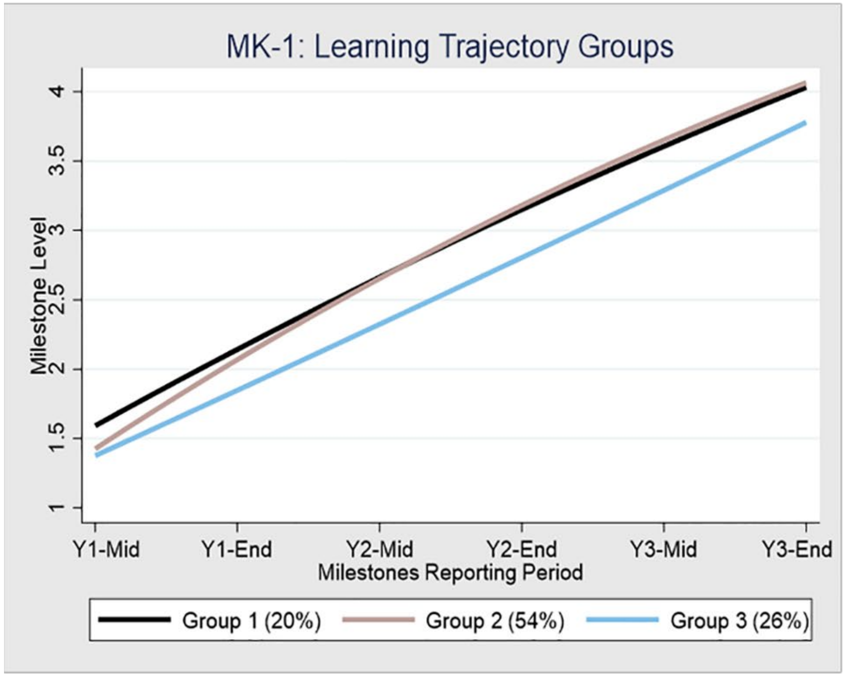

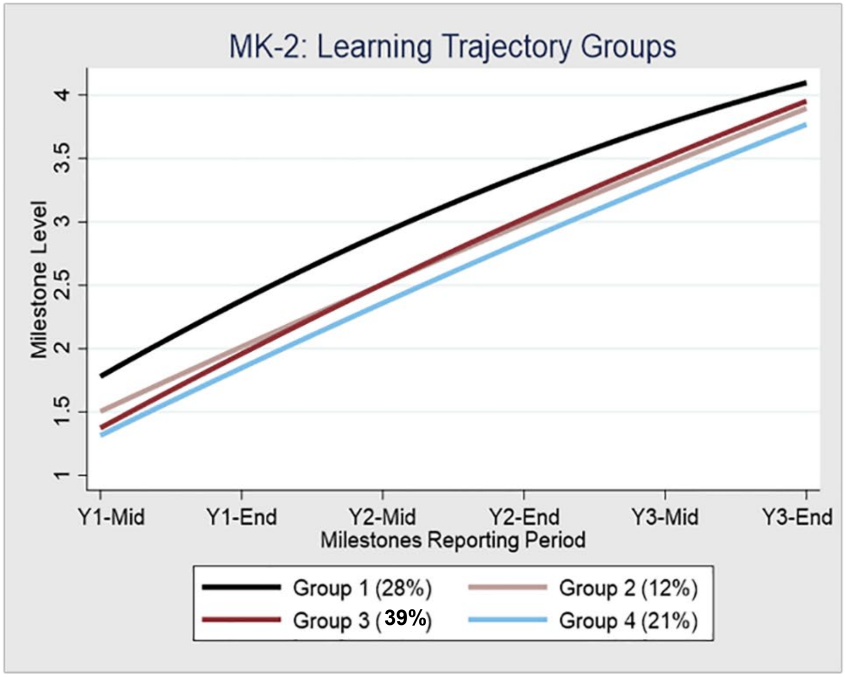

© 2021 Park YS et al. *JAMA Network Open.*
